# Optimization of isothermal amplification method for Mycobacterium tuberculosis detection and visualization method for fieldwork

**DOI:** 10.3906/sag-1910-6

**Published:** 2020-06-23

**Authors:** Esra AĞEL, Hasan SAĞCAN, İsmail CEYHAN, Rıza DURMAZ

**Affiliations:** 1 Materials Institute, TÜBİTAK Marmara Research Center, Kocaeli Turkey; 2 Department of Medical Laboratory Techniques, Vocational School of Health Services, İstanbul Medipol University, İstanbul Turkey; 3 Department of Medical Microbiology, Atatürk Chest Diseases and Chest Surgery Training and Research Hospital, Ankara Turkey; 4 Department of Medical Microbiology, Faculty of Medicine, Ankara Yıldırım Beyazıt University, Ankara Turkey

**Keywords:** *Mycobacterium tuberculosis*, loop-mediated isothermal amplification, lateral flow dipstick, colorimetric detection, point of care

## Abstract

**Background/aim:**

Tuberculosis is still one of the most contagious diseases around the world. Key factors of tuberculosis control are rapid diagnostic, efficient treatment, and prevention of contamination by surveillance and monitoring. However, culture is the gold standard method for laboratory diagnosis of tuberculosis; the results are several weeks to obtain. In order to prevent contamination of tuberculosis, diagnosis must be made in short time and treatment should be started as soon as possible. The aim of this study is to optimize the loop-mediated isothermal amplification (LAMP) method, which provides a much faster and more sensitive result than the polymerase chain reaction (PCR) method and allows the replication of target nucleic acid sequences under isothermal conditions without the need for laboratory infrastructure.

**Materials and methods:**

Sputum samples were homogenized with 5% trypsin solution in CaCl2 to obtain DNA. DNA was purified using QIAGEN QIAamp DNA mini kit. LAMP primers were design using Primer explorer V5 program according to IS6110 gene of *Mycobacterium tuberculosis*. NEB Bst 3.0 DNA polymerase kit was used for LAMP reactions. Besides, LAMP reactions were compared with TaqMan based RT-PCR method using NEB’s Taq polymerase kit. Finally, for visualization of LAMP products, lateral flow dipsticks that produced by Milenia Biotec, colorimetric 2X LAMP master mix that produced by NEB and 2% w/v agarose gel electrophoresis methods were used.

**Results:**

Optimum amplification temperature for LAMP was found to be 71.4 °C. The detection limit of the method was 102 CFU/mL and sensitivity was determined 100% compared to five different *Mycobacterium* species.

**Conclusion:**

The current study indicated that the LAMP-LFD and colorimetric LAMP protocol optimized with sputum samples can be reliable used as a rapid, sensitive and specific assay in the diagnosis of tuberculosis in the field.

## 1. Introduction

Tuberculosis caused by *Mycobacterium tuberculosis* is spread from person to person through respiratory fluids and air [1]. *M. tuberculosis* has a low infective dose; just below the 10 bacilli (1 to 200 bacilli) are enough for infection [2]. Tuberculosis comes right after HIV for infection-caused deaths [3]. According to the World Health Organization (WHO) reports, 1/3 of the world population today is already infected with tuberculosis. It was estimated that 10 million people developed tuberculosis and 1.6 million of these infected people died because of tuberculosis in 2017. About 1 million tuberculosis cases are seen in children. In Turkey, 12,046 new tuberculosis patients emerged in 2017 [4]. 

Key factors for the control of tuberculosis are a rapid diagnosis, effective treatment, and preventing transmission with scanning. The gold standard method for laboratory diagnosis of tuberculosis is culture method [5]. However, getting the results takes a few weeks long. Moreover, sensitivity of the microscopic examination following the Ziehl–Neelsen staining is low. For preventing tuberculosis spread, it is critical to diagnose the disease within 1 or 2 days and start the treatment immediately [6]. Therefore, there is a need for quick, sensitive, reliable, point-of-care, and economical methods for the laboratory diagnosis of tuberculosis. 

Nucleic acid amplification is one of the most effective methods for detecting infectious diseases and genetic differences [7,8]. Various amplification methods have been developed in addition to commonly used polymerase chain reaction (PCR)-based diagnostic methods [9]. One of these is loop mediated isothermal amplification (LAMP) method [10]. This method is based on DNA synthesis at an isothermal condition using DNA polymerase enzyme with high strand displacement activity and 4 primers recognizing 6 different regions in the target sequence with its unique design [11]. 

There are different methods for the detection of LAMP products. LAMP products can be displayed by methods commonly used in the detection of PCR products, such as gel electrophoresis, methods for detecting turbidity caused by reaction by-products, detection methods by varying the pH of reaction, and the use of lateral flow test strips [12,13].

Diagnostic technologies which are required laboratory infrastructure are less preferred due to reasons such as the high cost of methods, need for specialized teams, and the length of time [14]. However, the use of lateral flow immunoassays (LFIA), which are suitable for point of care diagnostic, has become widespread with the help of diagnostic and biosensor technologies. These recent developments have brought lateral flow immunoassays to a central position in diagnostic technologies. Briefly, lateral flow immunoassays are becoming increasingly widespread due to their versatility [15]. Lateral flow immunoassays are commonly used to detect biological agents and pathogens as well as fields such as medicine, veterinary medicine, and food [16].

In recent years, diagnostic technologies are in the direction of tests that require serious laboratory infrastructure and complex detection methods to point-of-care diagnostic methods [17]. The LAMP method is preferred for use in this study for *Mycobacterium tuberculosis* diagnosis because it is a relatively simple, rapid, highly specific method and does not require complex laboratory equipment [18]. The purpose of this study was to optimize the LAMP method for rapid and accurate diagnosis of tuberculosis as point-of-care testing. 

## 2. Materials and methods

### 2.1. Samples and DNA extraction method

Acid-fast bacilli (AFB) negative sputum, standard *Mycobacterium tuberculosis* H37Rv strain and sputum samples from 93 patients (68 culture positive, 25 culture negative) which was used in TÜBİTAK project (115R002) were provided by Atatürk Chest Diseases and Chest Surgery Training and Research Hospitals. The samples were decontaminated using the NALC-NaOH method. The sputum smears were analyzed for acid-fast bacilli using Ziehl–Neelsen staining and cultured on the LJ medium for 6–8 weeks. Standard strain grown on Lowenstein-Jensen medium was homogenized in phosphate buffer saline (PBS, pH:6.8) and its density was adjusted to McFarland 1, which is equivalent to 1.97 × 106  colony forming units (CFU)/mL and this density was used as main stock [19]. AFB negative sputum samples were spiked with main stock and its serial dilution from 1/10 to 1/100000. A nonspiked sputum sample was used for negative control. All sputum samples were homogenized with 5% trypsin (pH:8) which was dissolved in CaCl2 (25 mg/L) [20]. DNA was purified using QIAGEN QIAamp DNA mini kit, according to the manufacturer’s instructions. The purity and quality of DNA were controlled using Implen NanoPhotometer (Implen GmbH, Germany). The extracted DNA was stored at –20 °C until used. 

### 2.2. Optimization of LAMP reaction

*M. tuberculosis* specific LAMP primers were designed using PrimerExplorer V5 according to *IS6110* gene (GenBank accession no: X17348). Details of the primers were shown in Table 1. Furthermore, a probe of 21 bp was selected in the target sequence outside the primer region. The primer sequences given by the V5 program were checked to confirm whether there was any homology among the *Mycobacterium* species other than *M. tuberculosis.*

**Table 1:** Mycobacterium tuberculosis IS6110 gene-specific LAMP primers.

Primers	Sequences	Lenght
F3	CGAATTGCGAAGGGCGA	17
B3	GTAGGTCGATGGGGCGA	17
FIP	ACCGTTAATTAGCGTGCTGGCC-ATTTTAAAGACCGCGTCGGC	42
BIP	GATCATCAGGGCCACCGCGAG-CGGTCAGCTGTGTGCAGAT	40

Initial conditions of LAMP reaction which was performed in 25 µL, containing 0.2 µM each outer primers (F3 and B3), 1.6 µM each inner primers (FIP and BIP), 20 mM Tris-HCl (pH: 8.8), 10 mM KCl, 10 mM (NH4)2SO4, 9 mM MgSO4, 1.4 mM dNTP, 8 U *Bst* polymerase, and 5 µL DNA sample. For optimization of LAMP, reactions were carried out 0.1–0.5 µM each outer primers, 0.4–2.0 µM each inner primers, 2–10 mM MgSO4, 0.2–2 mM dNTP, and 1–10 U *Bst* polymerase. For optimization of temperature and time, reactions were incubated at 60–74 °C for 30–60 min and enzyme denaturation incubated at 80 °C for 10 min. 

### 2.3. Visualization of LAMP product

LAMP products were visualized using lateral flow dipstick and colorimetric LAMP kit, and gel electrophoresis methods. A FAM labelled-probe (FAM-5’ CATCAGGGCCACCGCGAGGGC-3’) was used for LFD detection which was performed according to the manufacturer’s instructions. After the LAMP reactions, 20 pmole FAM-labelled probes were added and incubated for 5 min at 65 °C. At the end of hybridization, 8 µL hybridized product was transferred to 100 µL assay buffer. Finally, a test strip was dipped into the final product and the result was observed in 5 min. For the colorimetric assay, NEB Warmstart colorimetric LAMP 2X master mix kit was used and the process was performed according to the manufacturer’s instructions. LAMP products were also analyzed by 2% w/v agarose gel electrophoresis and observed by Avegene UV-transilluminator.

### 2.4. Sensitivity of LAMP assay

The sensitivity of LAMP methods was compared with the RT-PCR assay using sputum samples spiked by *M. tuberculosis* with different densities ranging from 101 to 105 CFU/mL, each density was prepared in two samples.

### 2.5. Specificity of LAMP assay

Specificity assay of LAMP and RT-PCR methods was tested using the 5 different *Mycobacterium* other than tuberculosis (MOTT) species which were *M. avium*, *M. kansasii*, *M. intracellulare*, *M. gordonae*, and *M. fortiutum*. 

### 2.6. RT-PCR Assay

Real-time PCR (RT-PCR) method described by Desjardin et al. (1996) was used as a reference test. IS6 (5’-GGCTGTGGGTAGCAGACC-3’), IS7(5’-CG GGTCCAGATGGCTTGC-3’) primers and IS6110 Taqman probe (5’-(FAM)- TGTCGACCTGGGCAGGGTTCG-(TAMRA)-3’) were used in RT-PCR [21]. RT-PCR was carried out in 25 µL, containing 0.2 mM dNTP, 0.2 µM IS6 and IS7, 0.1 µM probe, 1 U Taq polymerase, 5 mM MgCl2, and 5 µL DNA sample. PCR reaction was performed with BioRad CFX96 with following conditions: 50 °C for 2 min, 95 °C for 5 min, and 45 cycles of 94 °C for 30 s and 68 °C for 1 min. 

## 3. Results

### 3.1. Primer design

The LAMP primers sequences given by the V5 program were checked to confirm that there was no homology against any species except *M. tuberculosis*. 

### 3.2. Optimization of the LAMP assay

**Figure 1:**
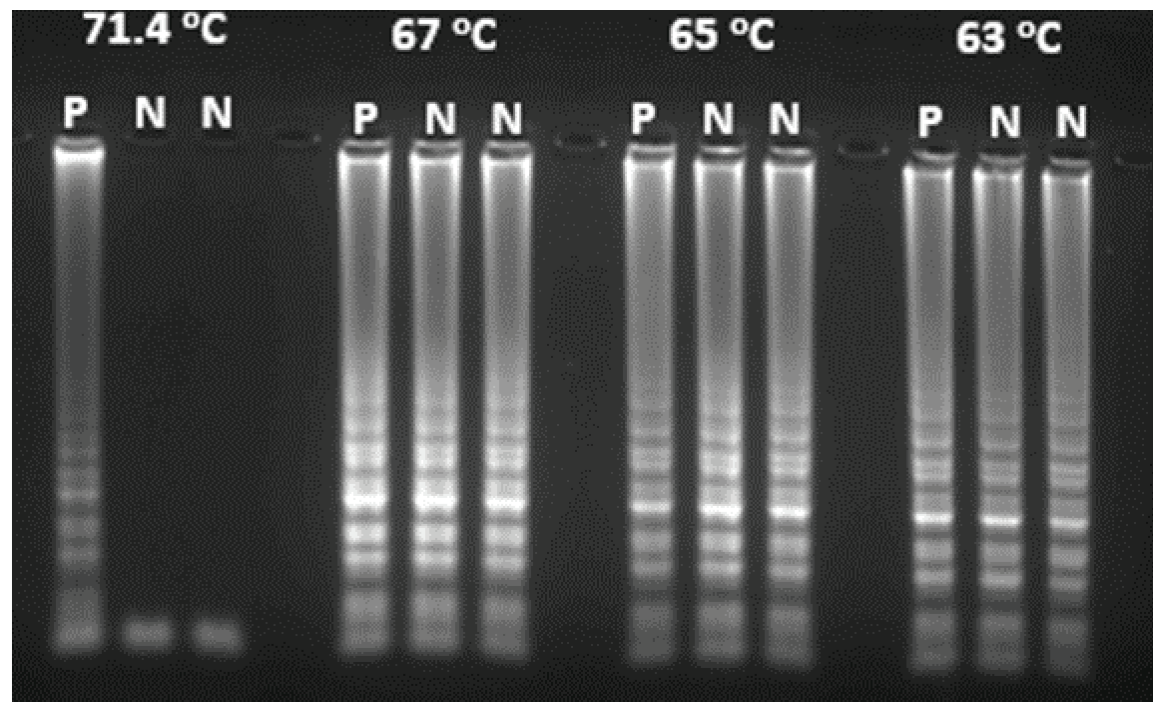
Visualization by agarose gel electrophoresis of LAMP assays carried on different temperatures. P: Positive control included M. tuberculosis with a concentration of 105 cfu/mL, N: negative control prepared from AFB negative sputum.

By changing the concentration of each component and amplification condition, the optimum conditions for LAMP amplification were determined to be 60 min at 71.4 °C. The lower temperatures such as 63 °C, 65 °C, and 67 °C yielded false-positive results in negative controls (Figure 1). The optimum concentrations were determined as 0.4 mM for dNTPs, 2 U for Bst polymerase, 5 mM for MgSO4, 0.2 µM for F3 and B3 primers, 0.8 µM for FIP and BIP primers (Table 2). 

**Table 2:** M. tuberculosis detection performance of LAMP and RT-PCR methods. PPV: Positive predictive value, NPV: Negative predictive value, CI: Confidence interval

Methods	Sensitivity (%)	Specificity (%)	NPV (%)	PPV (%)
LAMP (95% CI)				
· Culture Method	92.75% (83.89- 97.61%)	91.67% (73.00- 98.97%)	81.48% (65.22- 91.17%)	96.97% (89.45-99.18%)
RT-PCR (95% CI)				
· Culture Method	83.82% (72.90- 91.64%)	88.00% (68.78- 97.45%)	66.67% (53.32- 77.79%)	95.00% (86.74-98.22%)

### 3.3. Visualization of LAMP product

**Figure 2:**
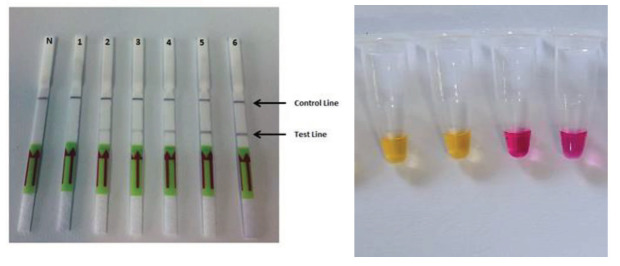
Visualization by lateral flow dipstick (a) and colorimetric method (b) of the optimized LAMP assay. a) N: negative control, 1: 101 cfu/mL, 2: 102 cfu/mL, 3: 103 cfu/mL, 4: 104 cfu/mL, 5: 105 cfu/mL, 6: 106 cfu/mL. b) Yellow color indicates a positive LAMP assay.

The LAMP assay performed after optimization did not give any false positive results with the visualization of gel electrophoresis, lateral flow dipstick, and colorimetric methods. In gel electrophoresis methods, ladder-like band formation was observed on the samples spiked by *M. tuberculosis* samples, but only negative control samples were not observed ladder-like band formation at 71.4 °C. In the lateral flow dipstic, the test line was observed only in samples spiked with *M. tuberculosis* and there was no test line in the negative control line (Figure 2a). In the colorimetric method, the yellow color was observed in only the samples spiked with *M. tuberculosis*, and but in negative controls, it was not detected any color change (Figure 2b).

### 3.4. Sensitivity of LAMP assay

Serial dilutions between 105 CFU/mL and 101 CFU/mL of the *M. tuberculosis* samples were prepared in sputum samples to measure the sensitivity of LAMP assay. As a result, it was determined that the minimum amount of *M. tuberculosis* for the positive results, or in other words the detection limit of the method, was 102 CFU/mL. The optimized LAMP assay yielded positive results in all of the 8 samples spiked with *M. tuberculosis* strain with concentrations of ≥102 CFU/mL. The same samples were also studied with the RT-PCR method and its detection limit and sensitivity were 101 CFU/mL and 100%, respectively. 

### 3.5. Specificity of LAMP assay

The specificity of LAMP primers was checked on *M. tuberculosis*, *M. avium*, *M. kansasii*, *M. intracellulare*, *M. gordonae*, *M. fortiutum*, and negative sputum samples. It was determined that the LAMP primers did not give a positive result with any of the non-*M. tuberculosis* species tested.

As a result of the study performed with 93 patient’s sputum samples (68 culture positive, 25 culture negative), 64 samples were LAMP positive, 22 samples were LAMP negative, 2 samples were false-positive, and 5 samples were false negative. Of 68 culture positive samples, 57 were RT-PCR positive, 22 of 25 culture negative samples were RT-PCR negative. Three samples were false positive, and 11 samples were false negative in the RT-PCR method. The results of statistical analysis are given in Table 2.

## 4. Discussion

Loop mediated isothermal amplification is a fast method that replicates the target nucleic acid sequences under isothermal conditions at a constant incubation temperature. The LAMP method is more specific than the other PCR methods because it uses 4 or 6 different primers that specifically recognize 6 to 8 different regions of the target gene [22,23]. Generally, 50 to 100 times more amplicons are produced in the LAMP method than other types of PCR [24]. Although there are many different isothermal amplification methods besides LAMP, most of these methods are not suitable for the amplification of large DNA sequences [25]. However, the LAMP method can amplify a large DNA target more specifically and precisely [26,27]. Moreover, the LAMP method can be easily integrated into microchip diagnostic devices (Lab-on-a-chip), sensor-based devices, and point-of-care diagnostic devices [28,29]. Therefore, in the current study, optimization of the LAMP method, which became popular in the last decade for the detection of *M. tuberculosis,* was performed to use it for point-of-care testing. 

In this study, the optimum temperature of LAMP assay for *M. tuberculosis* detection was found at 71.4 °C, which differed from the previous studies. Previous studies reported that the optimal amplification temperature of LAMP assay for *M. tuberculosis* detection was 63 °C [30]. The reason for the low amplification temperature in other studies might be the use of betaine [31]. However, in our study, the use of betaine was found to cause false positivity at low temperature. 

Due to the 50 to 100 times more amplicons produced in the LAMP method than other types of PCR, it is expected that the sensitivity of this method might be higher than other methods based on PCR. However, in this study, the sensitivity of the LAMP method optimized for the diagnosis of *M. tuberculosis* was 102 CFU/mL, and the sensitivity by the RT-PCR method was 101 CFU/mL. Kaewphinit et al. [32] reported that combining the gold nanoparticle with the LAMP method for tuberculosis detection reduced its detection limit to 101 CFU/mL. It seems that combining the gold nanoparticle with the LAMP method is not useful in detecting LAMP products since the inhibition can also occur when trying to improve the detection limit with the gold nanoparticle.

Many methods such as gel electrophoresis, turbidity detection, calcein staining, lateral flow test strips (LFD), the colorimetric method can be used for the detection of LAMP products [33]. *M. tuberculosis* detection with LAMP-LFD or colorimetric method indicated high sensitivity and relatively shorter analysis time when compared to conventional PCR and real-time PCR [34–36]. In this study, gel electrophoresis, lateral flow test strips, and Warmstart colorimetric LAMP 2X master mix kit were used. Methods based on precipitation detection and colorimetric methods using pH-sensitive dye are widely used to visualize products in the LAMP method [37]. As the amplicon is formed in the LAMP studies by 100 times more than RT-PCR and conventional PCR, the resulting precipitate and reaction can be detected by the naked eye with the changing pH [37]. Due to this advantage of the method, complex devices and laboratory dependence are eliminated. In this study, analysis results were evaluated using the NEB Warmstart colorimetric LAMP 2X master mix kit. It was observed, similar to other studies, that this method gave false positives and/or negativity results because of differences in pH from the sample or DNA isolation [38]. On the other hand, the LFD used in the current study was not affected by pH changes and it did not yield false positive or negative results. Prompamorn et al. [39] reported that using LFD increased sensitivity 3–10 times. Nevertheless, the sensitivity of LFD and gel electrophoresis method was detected to be 102 CFU/mL in this study. Consequently, it was shown that there was no positive effect of LFD to the sensitivity of the method. However, with the use of LFD, the duration of electrophoresis was shortened, and it has gained about an hour. In addition, the use of carcinogen (EtBr) used during gel electrophoresis was discontinued. Finally, the LFD method was more advantageous in terms of cost and ease of use, since there was no need for imaging devices used in the gel electrophoresis method [40]. Due to primers that specifically recognize 6 to 8 different regions of the target gene, used in the LAMP method, it is expected that the specificity of this method might be higher than other methods based on PCR. In this study, amplification of DNA was observed only with *M. tuberculosis* and there was no amplification in 5 different *Mycobacterium* species. Our results were consistent with those of a previous study which used the LAMP method to detect *M. tuberculosis* [41].

As a result of the study performed with 93 patient’s sputum samples (68 culture positive, 25 culture negative), an optimized LAMP method was found to be suitable for *M. tuberculosis* detection. When evaluated according to the results of culture method, the sensitivity and specificity of the LAMP method were found to be 92.75% (83.89–97.61%) and 91.67% (73.00–98.97%). The sensitivity and specificity of the RT-PCR method were found to be 83.82% (72.90–91.64 %) and 88.00% (68.78–97.45%). In the study performed by Nakiyingi et al., sensitivity and specificity of the LAMP assay were reported as 55.4% (44.1–66.3%) and 98.0% (94.3–99.6%) [42]. Even though the specificity of our method is lower than that of the referenced paper, it may still be preferred according to sensitivity value. Moreover, both the specificity and sensitivity are higher than the RT-PCR method used in this study, which emphasizes the LAMP method for the detection of *M. tuberculosis* with DNA amplification. 

In conclusion, in this study, a loop mediated isothermal amplification method was optimized for the detection of* M. tuberculosis* at level of 102 CFU/mL. The visualization of the LAMP products was shown to be feasible independently from the laboratory infrastructure using lateral flow test strip method and colorimetric LAMP method.

## Acknowledgment

This research was supported by TÜBİTAK (The Scientific and Technological Research Council of Turkey) under the Project 1003 115R002 entitled ‘‘Development of Integrated Microfluidic Chip Based Diagnostic Kit for Sensitive and Rapid Diagnosis of Tuberculosis Infection’’. Any opinions, findings, and conclusions expressed in this material are those of the authors and do not necessarily reflect the views of TÜBİTAK. We are grateful to TÜBİTAK for financial support.
